# Middle-Aged Indians with Type 2 Diabetes Are at Higher Risk of Biological Ageing with Special Reference to Serum CDKN2A

**DOI:** 10.1155/2020/7569259

**Published:** 2020-03-23

**Authors:** Joyita Banerjee, Yogita Dhas, Neetu Mishra

**Affiliations:** Symbiosis School of Biological Sciences (Formerly Symbiosis School of Biomedical Sciences), Symbiosis International (Deemed University), Lavale, Pune, India

## Abstract

Sedentary lifestyle and high visceral adiposity have elevated the risk of type 2 diabetes (T2DM) among Indians at younger age. In this study, we aimed to investigate the association of oxidative stress and chronic inflammatory mediators with ageing with special reference to the biological ageing marker cyclin-dependent kinase inhibitor 2A (CDKN2A) among middle-aged (31-50 years) Indian healthy and T2DM subjects. Malondialdehyde (MDA), oxidized LDL (oxLDL), interleukin-6 (IL-6), interleukin 1*β* (IL-1*β*), tumor necrosis factor *α* (TNF-*α*), monocyte chemoattractant protein-1 (MCP-1), and CDKN2A were measured in T2DM patients (*n* = 80) and controls (*n* = 80) aged 31-50 years, further grouped into G1: 31-40 years and G2: 41-50 years. IL-6, TNF-*α*, MCP-1, and CDKN2A showed a significant association with ageing among both T2DM patients and controls. But the strength of the association of MCP-1 and CKDN2A with ageing was significantly stronger in T2DM patients than the controls. All the oxidative stress and proinflammatory mediators showed nonsignificant associations with CDKN2A in the controls. However, IL-6, TNF-*α*, and MCP-1 showed a strong association with CDKN2A in T2DM patients. An increased risk of high levels of CDKN2A was found in G1 T2DM patients (OR: 3.484 (95% CI: 1.246-9.747) *p* = 0.017) and G2 T2DM patients (OR: 5.000 (95% CI: 1.914-13.061), *p* = 0.001) with reference to the respective control groups. Our study reveals that the middle-aged Indians with T2DM are at higher risk of biological ageing. The development of T2DM is more common among middle-aged Indians. T2DM may exacerbate the ageing process and may subsequently predispose Indians to various age-related complications at a much early age.

## 1. Introduction

The current decade has experienced a growing prevalence of diabetes worldwide. According to the International Diabetes Federation (IDF) 2017 reports, China is having the highest number of diabetic individuals in the world (approximately 114.4 million) followed by India with more than 72.9 million of diabetics [1]. The rapid urbanization, sedentary lifestyle, high-calorie diet, visceral adiposity, and high genetic predisposition have been identified as the major factors that elevate the risk of type 2 diabetes mellitus (T2DM) among Indians at a much younger age and at a lower body mass index (BMI) than the western population [2]. Various population-based studies reported that the average onset of T2DM among Indians is gradually increasing in the age groups below 50 years of age [2, 3].

The major pathological characteristics of T2DM are chronic hyperglycemia, dyslipidemia, and increased insulin resistance, which induce a plethora of metabolic and molecular alterations, eventually leading to the development of diabetes-associated vascular complications [4]. Chronic hyperglycemia elevates the levels of oxidative stress in the body and concomitantly activates various stress-related pathways such as the polyol, advanced glycation end product formation, activation of protein kinase C, and nuclear factor-kappa B (NF-*κ*B) pathways [5]. The previous studies reported that malondialdehyde (MDA) and oxidized LDL (oxLDL), as the markers of lipid peroxidation, appear to increase with T2DM pathogenesis [6, 7]. Another critical component involved in the pathogenesis of T2DM is the underlying chronic low-grade systemic inflammation, also known as metaflammation, induced due to the metabolic alterations stimulated by a surplus of nutrients [8]. The chronic low-grade inflammation is also responsible for islet inflammation contributing to *β*-cell dysfunction coupled with the downregulation of insulin gene transcription and *β*-cell apoptosis [9]. Several studies reported that the low-grade inflammation is characterized by the overexpression of proinflammatory cytokines, such as tumor necrosis factor *α* (TNF-*α*), interleukin-6 (IL-6), interleukin 1*β* (IL-1*β*), and monocyte chemoattractant protein-1 (MCP-1), in the obesity-induced insulin resistance, reduced insulin sensitivity, and pathogenesis of T2DM [10, 11].

However, both oxidative stress and chronic low-grade inflammation are the most quintessential aetiologies that underlie the molecular or cellular ageing [12]. The higher level of free radicals and chronic inflammation cause cellular damages, and both are the leading causes for loss of tissue or organ functionality, which eventually culminates in system dysfunction [13]. Moreover, recent researches emphasized that the presence of chronic hyperglycemia along with insulin resistance affects the normal ageing process and may be the reason for accelerating ageing [14, 15]. The previous studies have shown that diabetes induces premature cellular senescence in various cell types such as *β*-cells, endothelial cells, and cardiomyocytes [14–16].

Cellular senescence is an important mechanism that causes an irreversible proliferative arrest of the cell cycle at the G1 phase and acts as the prominent driver of the ageing process [17]. There are certain biomarkers of cellular senescence such as telomere shortening and cyclin-dependent kinase inhibitor 2A (CDKN2A) which reflect the alterations in molecular or cellular processes intrinsically associated with biological ageing [18, 19]. CDKN2A is a more dynamic marker of senescence which encodes two cell cycle inhibitors p16^Ink4a^ and p19^Arf^ [20]. Both p16^Ink4a^ and p19^Arf^ are expressed at high levels in senescent cells and are thought to play vital roles in the senescence process [20, 21]. In particular, p16^Ink4a^ blocks the cyclins and cyclin-dependent kinases (CDKs), prevents cell cycle progression, and has been identified as both the marker and the effector of senescence of *β*-cells [20]. On the other hand, p19^Arf^ stabilizes p53, another prominent marker of cellular senescence [21]. Moreover, CDKN2A/p16 is associated with establishing a permanent or full senescence process thus elucidating its crucial role in the ageing process [22].

Ageing is a continuous process in every individual. There are several population-based studies on ageing. Most of the findings on ageing have mainly focused on the elderly and geriatric population [7, 23]. There are only a handful of studies which addressed ageing in relatively young Indians with T2DM [19]. However, the dearth of information regarding the effect of T2DM-induced oxidative stress and low-grade inflammation on ageing has not been comprehensively explored among the relatively young Indian population. Moreover, there are hardly any studies which looked upon serum levels of biological ageing marker CDKN2A in this population. Hence, we aimed to investigate the association of oxidative stress and chronic inflammatory mediators with ageing with special reference to CDKN2A among middle-aged (31-50 years) Indian healthy and T2DM subjects.

## 2. Materials and Methods

### 2.1. Study Population and Study Design

A case-control study was conducted at the Symbiosis School of Biological Sciences, Symbiosis International (Deemed University), Pune, India, which enrolled subjects (*n* = 160), both males and females of age group 31-50 years. The subjects were grouped into type 2 diabetic or T2DM patients (*n* = 80), recruited from private diabetic clinics of Pune city, and the healthy controls (*n* = 80), which included willing locals of Pune city.

The middle-aged T2DM patients and controls were further grouped based on age groups as follows:

- Group 1 (early middle aged: 31-40 years): G1 T2DM patients (*n* = 40) and G1 controls (*n* = 40)
- Group 2 (late middle aged: 41-50 years): G2 T2DM patients (*n* = 40) and G2 controls (*n* = 40)

### 2.2. Inclusion and Exclusion Criteria of Study Subjects

Inclusion criteria of T2DM patients were as per the American Diabetes Association guidelines (2017) [24]. The controls were comprised of apparently healthy individuals with a subjective perception of good health, not on medications 2 to 3 months before the sample collection, and without diabetes and any major medical illness. The patients and controls were not on any antioxidant and anti-inflammatory therapies. Individuals suffering from common flu, fever, any chronic diseases like cancer, diabetic microvascular complications, diabetic macrovascular complications, neurodegenerative diseases, pregnant, and lactating women were excluded from the study.

### 2.3. Ethical Statement

The study protocol received approval of the Institutional Independent Ethics Committee and performed according to the ethical standards as laid down in the 1964 Declaration of Helsinki and its later amendments. The written informed consent was obtained from each subject before the initiation of the study.

### 2.4. Demographic Variables, Anthropometric Data, and Biochemical Methods

All the subjects completed the study questionnaire, blood pressure, and anthropometric measurements as described previously [25]. The anthropometric data included measuring height, weight, waist circumference (WC), and hip circumference (HC). 10 mL venous blood sample of each subject was collected after 10-12 hr overnight fasting, under all aseptic conditions by the phlebotomist. The blood samples were processed for plasma and serum separation and were stored at -80°C in separate aliquots. All the tests were performed within one month from the date of sample collection. The biochemical investigations included fasting plasma glucose (FPG) (glucose oxidase-peroxidase method), hemoglobin A_1C_ (HbA_1c_) estimated by the immunoturbidimetric method (Randox Laboratories), and lipid profile analysis, which included estimation of total serum cholesterol (TC), serum high-density lipoprotein (HDL-c), and serum triglycerides (TG) by enzymatic methods. Low-density lipoprotein (LDL-c) was calculated using Friedewald's formula [26]. The fasting plasma insulin (FPI) was measured by sandwich ELISA (Invitrogen, USA). The insulin resistance (HOMA-IR) was calculated by the homeostatic model assessment (HOMA) method [27].

### 2.5. Determination of Oxidative Stress Markers, hs-CRP, and Proinflammatory Cytokines

Plasma MDA was measured by the thiobarbituric acid-reacting substance method (TBARS) as described by Placer et al. [28]. Serum oxLDL was quantitatively determined by sandwich ELISA (catalog: SEA527Hu, USCN, Cloud-Clone, USA). The assay was performed as per the manufacturer's protocol, and the absorbance was read on the ELISA plate reader (Biotek, ELx800) at 450 nm.

Next, the quantitative determination of serum high-sensitivity C-reactive protein (hs-CRP), a well-recognized inflammatory marker, was carried out by sandwich ELISA (catalog: CR120C, CalBiotech, USA). The subjects with hs‐CRP level > 10 mg/L were excluded from the study as it represented the case of acute inflammation [29].

The serum concentrations of proinflammatory cytokines IL-6, IL-1*β*, TNF-*α*, and MCP-1 were measured by the Cytometric Bead Array (CBA) method (catalog: 558276 (IL-6 flex set), 558279 (IL-1*β* flex set), 560112 (TNF-*α* flex set), 558287 (MCP-1 flex set), 558264 (CBA buffer kit), BD Biosciences, USA), using flow cytometry (BD FACSCalibur). The assay was performed according to the manufacturer's protocol. Briefly, each capture bead of each cytokine in the BD CBA Human Soluble Protein Flex Set System has a distinct fluorescence and is coated with a capture antibody specific for a soluble protein. The detection reagent is a mixture of phycoerythrin- (PE-) conjugated antibodies, which provides a fluorescent signal in proportion to the amount of bound analyte. Undetectable analyte levels in the serum were considered 0 pg/mL and included in the statistical analysis.

### 2.6. Determination of Serum CDKN2A

The quantitative determination of serum CDKN2A was carried out by sandwich ELISA by following the manufacturer's protocol (catalog: SEA527Hu, USCN, Cloud-Clone, USA). The concentration of CDKN2A (ng/mL) in the serum samples was measured spectrophotometrically at a wavelength of 450 nm. The concentration of CDKN2A in the sample was then determined by comparing the absorbance of the sample to the standard curve.

### 2.7. Statistical Analysis

The statistical analysis was done by using the Statistical Package for the Social Science (SPSS) version 16.0, IL, USA. Assumptions of normality of data and homogeneity in variances were determined by Shapiro-Wilk's test of normality and Levene's test of equality of variances, respectively. Data were represented as the median and interquartile range (IQR) (Q1-Q3). Comparisons between groups were performed using the Mann–Whitney *U* test or Kruskal-Wallis test or Independent *t*-test or Chi-square test as appropriate. The cut-off value of each marker was set at the 75^th^ percentile of the controls. Based on cut-offs, the levels of markers were categorized into low and high levels. Logistic regression was carried out to predict risk of ageing in the presence of T2DM. The statistical significance was set at *p* < 0.05.

## 3. Results

The baseline characteristics of the study subjects are shown in Table 1. BMI of G1 T2DM patients was significantly higher (*p* < 0.01) than that of G1 controls, whereas G2 T2DM patients had increased central obesity (WC: *p* < 0.01; WHR: *p* < 0.05) as compared to G2 controls. In glycemic indices, FPG, HbA_1c_, and HOMA-IR were found to be significantly different (p < 0.01) in two age groups of T2DM when compared to their respective control groups. In the lipid profile, only TG was significantly higher (*p* < 0.05) in both the groups of T2DM as compared to controls. The cardiac risk marker hs-CRP was increased considerably in G1 T2DM patients, whereas senescence marker CDKN2A was significantly increased in G2 T2DM patients. However, a significant difference in CDKN2A was found between T2DM patients and controls in the G1 group by adjusting FPI (*p* = 0.001) and HOMA-IR (*p* = 0.005) by the analysis of covariance (ANCOVA).

The demographic characteristics and current medications taken by T2DM patients were presented in Supplementary material (see [Supplementary-material supplementary-material-1]). Majority of patients (G1: 55% and G2: 67.5%) were on metformin in combination with other class of hypoglycemic drugs.

**Figure 1:**
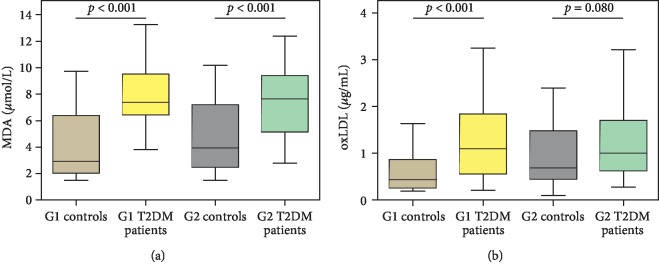
Oxidative stress markers in different study groups. (a) MDA (*μ*mol/L); (b) oxLDL (*μ*g/mL); G1: 31-40 years; G2: 41-50 years. Data are represented in the median and interquartile range (IQR); *p* value using the Mann–Whitney *U* test; MDA: malondialdehyde; oxLDL: oxidized LDL.

The median and IQR of the oxidative stress markers (MDA and oxLDL) are presented in Figure 1. MDA was significantly higher in both the age groups (G1 and G2) of T2DM patients as compared to their respective control groups. But oxLDL showed a significant difference between G1 T2DM patients and G1 controls only.

**Figure 2:**
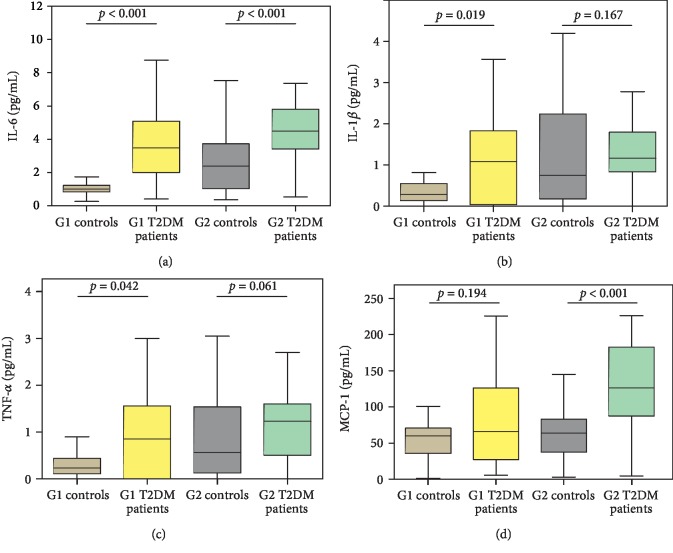
Proinflammatory cytokines in different study groups. (a) IL-6 (pg/mL); (b). IL-1*β* (pg/mL); (c). TNF-*α* (pg/mL); (d). MCP-1 (pg/mL); G1: 31-40 years; G2: 41-50 years. Data are represented in the median and interquartile range (IQR); *p* value using the Mann–Whitney *U* test; IL-6: interleukin-6; IL-1*β*: interleukin-1*β*; TNF-*α*: tumor necrosis factor *α*; MCP-1: monocyte chemoattractant protein-1.

Similarly, the median and IQR of proinflammatory cytokines (IL-6, IL-1*β*, TNF-*α*, and MCP-1) in different age groups are presented in Figure 2. The proinflammatory cytokines such as IL-6, IL-1*β*, and TNF-*α* are observed to be significantly higher in G1 T2DM patients, whereas IL-6 and MCP-1 increased substantially in G2 T2DM patients as compared to their respective controls.

The association of oxidative stress markers, proinflammatory cytokines, and senescence marker with ageing in T2DM and controls was tested using the Chi-square test of independent association (see Table 2). All the markers were categorized into two levels, low and high based on their cut-off values (MDA > 7.6 *μ*mol/L, oxLDL > 1.08 *μ*g/mL, IL‐6 > 3.04 pg/mL, TNF‐*α* > 0.88 pg/mL, IL‐1*β* > 1.16 pg/mL, MCP‐1 > 77.3 pg/mL, and CDKN2A > 4.46 ng/mL. Among oxidative stress markers, only oxLDL showed a significant association with ageing in controls, while this association was nonsignificant in T2DM patients. All the proinflammatory cytokines were significantly associated with ageing among controls, with IL-1*β* and TNF-*α* showing a highly significant association (*p* < 0.01). IL-6, TNF-*α*, and MCP-1 showed a significant association with ageing among T2DM patients. However, MCP-1 showed a stronger association (*p* < 0.01) with ageing in T2DM patients than in controls. Higher percentages of high CDKN2A levels were observed in T2DM patients than in controls. The association of CDKN2A with ageing was significant among controls (*p* < 0.05), but the strength of this association was significantly higher among T2DM patients (*p* < 0.01).

Similarly, we have further checked the association of the biological ageing markers, that is, CDKN2A with oxidative stress markers and proinflammatory cytokines (see Table 3). All the markers were categorized into low and high levels based on the same cut-offs as described in Table 2. All the markers showed nonsignificant associations with biological ageing marker CDKN2A in controls. However, IL-6, TNF-*α*, and MCP-1 were strongly associated with CDKN2A in T2DM patients, with MCP-1 showing a very high significant association (*p* < 0.001) and oxLDL showing a marginal significant association (*p* = 0.075).

We have also checked the differences in these markers according to the duration of diabetes (see Supplementary material [Supplementary-material supplementary-material-1]), which indicated that MCP-1 (*p* = 0.002) and CDKN2A (*p* < 0.001) significantly increased with the duration of diabetes. We also determined the effect of glycemia on these markers (see Supplementary material [Supplementary-material supplementary-material-1]), which showed that the senescence marker CDKN2A increased significantly (*p* = 0.020) with the poor glycemic control (HbA_1c_ > 7.5%). Most of the T2DM patients were on metformin, so a separate analysis of medications was not performed.

Next, we performed logistic regression to determine the risk of high levels of senescence marker CDKN2A in different study groups (see Table 4). CDKN2A was considered the dependent variable. G1 controls were considered the reference group for G2 controls and G1 T2DM patients, whereas G2 controls were taken as the reference group for G2 T2DM patients. An increased risk of high levels of CDKN2A was found in both the groups of the T2DM patients with respect to the control groups. Among controls also, an increased risk of high levels of CDKN2A among G2 controls was observed with respect to G1 controls. The odds of high levels of CDKN2A among G1 T2DM patients and G2 controls were 3.484 times and 2.829 times, respectively, that of the G1 controls. On adjusting the adiposity indicators such as BMI, WC, and WHR (model 1), the associations between T2DM and CDKN2A in both the age groups remained significant. However, on adjusting oxidative stress markers (model 2), proinflammatory cytokines (model 3), and both (oxidative stress and proinflammatory cytokines) (model 4), this association became nonsignificant in the G1 age group but resulted in a significant association in the G2 age group.

## 4. Discussion

The present study showed an increased risk of biological ageing as evidenced by high levels of CDKN2A in middle-aged T2DM Indians. Significant associations of proinflammatory cytokines with ageing were found in both T2DM patients and controls. Among oxidative stress markers, only oxLDL showed a strong association with ageing in controls. Serum CDKN2A was significantly associated with ageing both in T2DM patients and controls, but higher strength of association was found in T2DM patients than in controls. Serum CDKN2A also showed significant associations with proinflammatory cytokines in T2DM patients and not in controls. Both oxidative stress and proinflammatory markers were significantly increased in the early-middle-aged (31-40 years) T2DM patients as compared to controls.

Ageing and T2DM are uniquely complex in development and progression, but they unite in some common aetiologies in which oxidative stress and low-grade inflammation are the main key players. The accumulation of deficits over time due to increased reactive oxygen species (ROS) and chronic levels of inflammatory mediators leads to the acquisition of the senescence-associated secretory phenotype (SASP) and progressive decline in cellular processes which ultimately causes ageing [13, 17].

In this study, we did not find any significant association of oxidative stress markers with ageing in T2DM patients. The distributions of high levels of both MDA and oxLDL were similar in early-middle-aged and late-middle-aged T2DM patients. We speculated that diabetes might have resulted in the early rise of these markers among T2DM patients. Prior evidence also suggested that the presence of high glucose enhanced oxidative stress [30]. We found MDA, the end product of lipid peroxidation, to be significantly higher in T2DM patients of both age groups when compared to their respective controls, similar to our previous finding [25]. Serum oxLDL, a well-recognized marker of atherosclerosis, showed a significant difference between G1 T2DM patients and G1 controls, and a marginally significant difference between G2 T2DM patients and G2 controls. The oxLDL is formed due to the oxidation of LDL-c. Earlier study determined that diabetic status enhances the oxidation of LDL-c to oxLDL [31].

We found significant associations of proinflammatory cytokines such as IL-6, IL-1*β*, TNF-*α*, and MCP-1 with ageing among controls. All these cytokines except IL-1*β* showed a significant association with ageing in T2DM patients. MCP-1 had a stronger strength of association with ageing in T2DM patients than in controls. The chronic low-grade inflammation is a distinctive feature of ageing, widely known as inflammageing, based on the expansion of the network and remodelling theory of ageing and forms the mechanistic basis of various age-related diseases such as cancer, Alzheimer's disease, cardiovascular diseases, and T2DM [32]. Recent developments have pointed at the overlapping mechanisms of inflammageing with metaflammation, and the combined effects of these two pillars of inflammation affect T2DM patients more strongly [33].

We found significant differences in the proinflammatory markers, IL-6, IL-1*β*, and TNF-*α* between G1 T2DM patients and G1 controls. IL-6 and MCP-1 showed significant differences between G2 T2DM patients and G2 controls. The earlier reports revealed that hyperglycemia induces a proinflammatory response in immune cells [34]. Hyperglycemia enhances the production of ROS. Metabolic deregulation coupled with high ROS levels increases the activation of NLRP3 (NOD-, LRR-, and pyrin domain-containing protein 3) inflammasome and caspase 1; the former is considered the promising pharmacological target in T2DM pathogenesis [4, 5]. The activation of inflammasome elicits the production of mature IL-*β*. The autostimulation of IL-*β* through a vicious cycle further induces various inflammatory mediators such as IL-6, IL-8, TNF-*α*, and MCP-1 through the IL-1 receptor (IL-1R) [4].

Our study revealed that serum CDKN2A significantly increased in G2 T2DM patients than G2 controls. CDKN2A encoding p16^Ink4a^ is widely accepted as the marker of cellular senescence, highly expressed in senescent cells, and is considered an important tissue- and blood-based molecular biomarker of biological ageing [20, 35]. Liu and colleagues demonstrated that p16 expression was associated with IL-6 and was independent of gender and BMI [35]. The determination of CDKN2A in the blood was suggested to be more advantageous and dynamic over telomere length as the biomarker of human ageing [35]. We found a significant difference in the concentrations of CDKN2A between G1 T2DM patients and G1 controls by adjusting fasting insulin and HOMA-IR. Insulin resistance affects T2DM pathogenesis and plays a vital role in the ageing process [36, 37]. Recent report revealed that insulin resistance accelerated the appearance of the senescent cells [37]. In a similar context, Aguayo-Mazzucato et al. demonstrated that insulin resistance accelerated the expression of p16^Ink4a^ in *β*-cells [15]. In our study, there was a significant association of CDKN2A with ageing in T2DM patients and controls. However, the strength of this association was stronger in T2DM patients than in controls. Higher risk of high levels of CDKN2A was found in T2DM patients with respect to controls. The previous studies also reported a significant association of CDKN2A locus with T2DM [38, 39]. Further, we observed a marked increase in CDKN2A level with the increase in duration of diabetes. We speculated that ageing process worsens as the duration of diabetes increases. CDKN2A was also significantly associated with proinflammatory mediators in T2DM patients. Hyperglycemia induces senescence; the latter further increases the proinflammatory state with an overexpression of a variety of cytokines, chemokines, and other soluble factors [14, 33]. The effect of oral hypoglycemic agents (OHA) might have contributed in ameliorating the oxidative stress level with ageing. OHA, in particular metformin, has the ability to regulate oxidative stress by the activation of adenosine monophosphate- (AMP-) activated protein kinase and increases the production of antioxidant thioredoxin [40]. Metformin also exerts an anti-inflammatory role by negatively regulating NF-*κ*B pathway [41]. However, we speculated that the increase in the low-grade inflammation with ageing might be due to the presence of persistent hyperinsulinemia and effects of the partial normalization of glycemia [42].

Our study provides valuable insights into the associations of oxidative stress and low-grade inflammation with biological ageing in middle-aged healthy and T2DM subjects. The present study reveals that the presence of T2DM significantly elevates the risk of premature ageing and age-related complications. Indians tend to have high visceral adiposity and higher ratio of fat to lean mass at any BMI level coupled with sedentary lifestyles and genetic predisposition, which concomitantly increased the risk of developing cardiometabolic diseases at a much early age [43]. Our findings highlight the significance of biological ageing marker CDKN2A in the early- and late-middle-aged Indian population. Nevertheless, our study is a one-point single-centered study, so further longitudinal study is necessary to address the causality.

## 5. Conclusion

Our findings extend the notion that oxidative stress and chronic inflammation induced by T2DM may aggravate the natural ageing process and may be responsible for accelerating biological ageing in middle-aged Indians. Our study lays the basis of future prospective studies to check whether lifestyle modifications can counteract premature ageing and age-related complications in T2DM patients.

## Figures and Tables

**Table 1:** Baseline characteristics of the study population according to age groups.

Variables	Group 1 (31-40 years)			Group 2 (41-50 years)		
	G1 controls (*n* = 40)	G1 T2DM (*n* = 40)	*p* value	G2 controls (*n* = 40)	G2 T2DM (*n* = 40)	*p* value
Age (years)	34 (31-36)	35.5 (33.25-39)	0.024	45 (42-48)	46 (43-48.75)	0.287
Body weight (kg)	65.95 (58.18-75.55)	71.05 (65.73-79.85)	0.054	64.25 (54.48-69.8)	66.45 (63-75)	0.011
Height (cm)	162 (157-171)	163.28 (156.25-170.75)	0.635^a^	158.5 (150.25-165.75)	163 (152.88-168.75)	0.295^a^
BMI (kg/m^2^)	24.91 (22.79-26.77)	27.11 (24.6-28.87)	0.002	26.13 (21.47-28.1)	27.23 (24.75-29.64)	0.082
WC (cm)	90.5 (86-93.75)	93 (84.63-102.38)	0.173	90.5 (83.38-94.5)	95 (91-99.5)	0.002
HC (cm)	97 (90.25-101.38)	101.2 (95-105)	0.009	98 (92.5-104.88)	100.5 (97.13-104.38)	0.170
WHR	0.929 (0.899-0.978)	0.915 (0.88-0.953)	0.138	0.927 (0.856-0.957)	0.956 (0.903-0.979)	0.021
SBP (mmHg)	116.5 (110.6-122.89)	124.5 (120-130)	<0.001^a^	116 (110-129.75)	125 (120-135)	0.001^a^
DBP (mmHg)	76.75 (72.63-80)	80 (77.88-81.75)	0.008	78 (71.5-81)	80 (80-84.75)	0.010
FPG (mmol/L)	4.59 (4.25-4.97)	7.5 (5.77-9.08)	<0.001	4.54 (4.23-4.9)	7.38 (6.26-10.77)	<0.001
FPI (*μ*IU/mL)	13.1 (9.24-21.14)	25.26 (16.1-40.74)	<0.001	19.46 (10.66-28.97)	16.62 (12.44-28.27)	0.810
HOMA-IR	2.72 (1.9-3.96)	8.85 (5.17-14.59)	<0.001	4.08 (2.12-5.88)	5.22 (3.47-10.03)	0.003
HbA_1c_ (%)	4.9 (4.7-5.18)	7.07 (6.7-8.3)	<0.001	4.97 (4.71-5.2)	8.32 (6.88-8.7)	<0.001
Duration of diabetes (years)	—	2.75 (1.5-5)	—	—	6.75 (3-10.88)	—
TG (mmol/L)	1.23 (0.94-1.57)	1.41 (0.97-2.37)	0.038	1.03 (0.86-1.41)	1.37 (1-1.82)	0.018
TC (mmol/L)	4.07 (3.45-4.52)	4.16 (3.32-5.18)	0.233^a^	4.21 (2.87-4.8)	3.76 (3.42-4.42)	0.662^a^
HDL-c (mmol/L)	1.05 (0.93-1.25)	0.91 (0.8-1.09)	0.004	0.96 (0.88-1.15)	0.95 (0.87-1.14)	0.866
LDL-c (mmol/L)	2.34 (1.87-2.84)	2.48 (1.7-3.03)	0.501	2.55 (1.45-3.18)	2.18 (1.67-2.58)	0.169
hs-CRP (mg/L)	3.65 (1.93-5.7)	6.35 (3.85-8.4)	0.001	4.4 (2.5-6.03)	4.72 (3.95-6.31)	0.095
CDKN2A (ng/mL)	3.346 (2.49-4.07)	3.589 (2.41-5.8)	0.137	3.184 (1.51-4.93)	7.5 (4.26-12.15)	<0.001

Data are represented as median and interquartile range (IQR); *p* value using the Mann–Whitney *U* test; ^a^*p* value using Independent *t*-test (for normally distributed data); BMI: body mass index; WC: waist circumference; HC: hip circumference; WHR: waist-hip ratio; FPG: fasting plasma glucose; FPI: fasting plasma insulin; HbA_1c_: hemoglobin A_1C_; HOMA-IR: homeostatic model assessment-insulin resistance; TG: triglyceride; TC: total cholesterol; HDL-c: high-density lipoprotein cholesterol; LDL-c: low-density lipoprotein cholesterol; hs-CRP: high-sensitivity C-reactive protein; CDKN2A: cyclin-dependent kinase inhibitor 2A.

**Table 2:** Association of oxidative stress markers, proinflammatory cytokines, and CDKN2A with ageing among T2DM patients and controls using the Chi-square test.

Group	Markers	Level of markers	Age group		*χ* ^2^, df	*p* value
			G1 (31-40 years)	G2 (41-50 years)		
Controls (*n* = 80)	MDA (>7.6 *μ*mol/L)	Low	31 (77.5%)	29 (72.5%)	0.267, 1	0.606
		High	9 (22.5%)	11 (27.5%)		
	oxLDL (>1.08 *μ*g/mL)	Low	35 (87.5%)	25 (62.5%)	6.667, 1	0.010
		High	5 (12.5%)	15 (37.5%)		
	IL-6 (>3.04 pg/mL)	Low	36 (90%)	27 (67.5%)	6.050, 1	0.014
		High	4 (10%)	13 (32.5%)		
	IL-1*β* (>1.16 pg/mL)	Low	37 (92.5%)	25 (62.5%)	9.600, 1	0.002
		High	4 (10%)	16 (40%)		
	TNF-*α* (>0.88 pg/mL)	Low	36 (90%)	25 (62.5%)	8.352, 1	0.004
		High	4 (10%)	15 (37.5%)		
	MCP-1 (>77.3 pg/mL)	Low	35 (87.5%)	25 (62.5%)	6.667, 1	0.010
		High	5 (12.5%)	15 (37.5%)		
	CDKN2A (>4.46 ng/mL)	Low	33 (82.5%)	25 (62.5%)	4.013,1	0.045
		High	7 (17.5%)	15 (37.5%)		

T2DM patients (*n* = 80)	MDA (>7.6 *μ*mol/L)	Low	18 (45%)	17 (42.5%)	0.051, 1	0.822
		High	22 (55%)	23 (57.5%)		
	oxLDL (>1.08 *μ*g/mL)	Low	19 (47.5%)	22 (55%)	0.450, 1	0.502
		High	21 (52.5%)	18 (45%)		
	IL-6 (>3.04 pg/mL)	Low	16 (40%)	6 (15%)	6.270, 1	0.012
		High	24 (60%)	34 (85%)		
	IL-1*β* (>1.16 pg/mL)	Low	21 (52.5%)	19 (47.5%)	0.200, 1	0.655
		High	19 (47.5%)	21 (52.5%)		
	TNF-*α* (>0.88 pg/mL)	Low	21 (52.5%)	12 (30%)	4.178, 1	0.041
		High	19 (47.5%)	28 (70%)		
	MCP-1 (>77.3 pg/mL)	Low	22 (55%)	8 (20%)	10.453, 1	0.001
		High	18 (45%)	32 (80%)		
	CDKN2A (>4.46 ng/mL)	Low	23 (57.5%)	10 (25%)	8.717,1	0.003
		High	17 (42.5%)	30 (75%)		

*χ* ^2^: Chi-square value; df: degrees of freedom; *p* value from the Chi-square test; each cell indicated count (% within age group); MDA: malondialdehyde; oxLDL: oxidized LDL; IL-6: interleukin-6; IL-1*β*: interleukin-1*β*; TNF-*α*: tumor necrosis factor *α*; MCP-1: monocyte chemoattractant protein-1; CDKN2A: cyclin-dependent kinase inhibitor 2A.

**Table 3:** Association of oxidative stress and proinflammatory cytokines with biological ageing marker CDKN2A among T2DM patients and controls using the Chi-square test.

Group	Markers	Level of markers	CDKN2A (>4.46 ng/mL)		*χ* ^2^, df	*p* value
			Low	High		
Controls (*n* = 80)	MDA (>7.6 *μ*mol/L)	Low	45 (77.6%)	15 (68.2%)	0.752, 1	0.386
		High	13 (22.4%)	7 (31.8%)		
	oxLDL (>1.08 *μ*g/mL)	Low	41 (70.7%)	19 (86.4%)	2.090, 1	0.148
		High	17 (29.3%)	3 (13.6%)		
	IL-6 (>3.04 pg/mL)	Low	44 (75.9%)	19 (86.4%)	1.051, 1	0.305
		High	14 (24.1%)	3 (13.6%)		
	IL-1*β* (>1.16 pg/mL)	Low	45 (77.6%)	15 (68.2%)	0.752, 1	0.386
		High	13 (22.4%)	7 (31.8%)		
	TNF-*α* (>0.88 pg/mL)	Low	44 (75.9%)	16 (72.7%)	0.084, 1	0.772
		High	14 (24.1%)	6 (27.3%)		
	MCP-1 (>77.3 pg/mL)	Low	46 (79.3%)	14 (63.6%)	2.090, 1	0.148
		High	12 (20.7%)	8 (36.4%)		

T2DM patients (*n* = 80)	MDA (>7.6 *μ*mol/L)	Low	12 (36.4%)	23 (48.9%)	1.245, 1	0.264
		High	21 (63.6%)	24 (51.1%)		
	oxLDL (>1.08 *μ*g/mL)	Low	13 (39.4%)	28 (59.6%)	3.160, 1	0.075
		High	20 (60.6%)	19 (40.4%)		
	IL-6 (>3.04 pg/mL)	Low	14 (42.4%)	8 (17%)	6.275, 1	0.012
		High	19 (57.6%)	39 (83%)		
	IL-1*β* (>1.16 pg/mL)	Low	19 (57.6%)	21 (44.7%)	1.289, 1	0.256
		High	14 (42.4%)	26 (55.3%)		
	TNF-*α* (>0.88 pg/mL)	Low	19 (57.6%)	13 (27.7%)	7.230, 1	0.007
		High	14 (42.4%)	34 (72.3%)		
	MCP-1 (>77.3 pg/mL)	Low	22 (66.7%)	8 (17%)	20.388, 1	<0.001
		High	11 (33.3%)	39 (83%)		

*χ* ^2^: Chi-square value; df: degrees of freedom; *p* value from the Chi-square test; each cell indicated count (% within age group); MDA: malondialdehyde; oxLDL: oxidized LDL; IL-6: interleukin-6; IL-1*β*: interleukin-1*β*; TNF-*α*: tumor necrosis factor *α*; MCP-1: monocyte chemoattractant protein-1; CDKN2A: cyclin-dependent kinase inhibitor 2A.

**Table 4:** Risk (OR) of high levels of CDKN2A in different study groups.

Senescence marker CDKN2A (ng/mL)	OR (95% CI) (*p* value)		
	G2 controls (reference: G1 controls)	G1 T2DM patients (reference: G1 controls)	G2 T2DM patients (reference: G2 controls)
Unadjusted	2.829	3.484	5.000
	(1.003-7.977)	(1.246-9.747)	(1.914-13.061)
	(0.049)	(0.017)	(0.001)

Model 1	3.648	3.329	5.654
	(1.190-11.183)	(1.121-9.880)	(1.873-17.064)
	(0.024)	(0.030)	(0.002)

Model 2	3.812	3.683	8.754
	(1.251-11.620)	(0.996-13.627)	(2.520-30.405)
	(0.019)	(0.051)	(0.001)

Model 3	3.221	3.212	4.356
	(1.036-10.013)	(0.898-11.493)	(1.374-13.811)
	(0.043)	(0.073)	(0.012)

Model 4	4.322	3.258	7.480
	(1.273-14.681)	(0.662-16.042)	(1.744-32.087)
	(0.019)	(0.146)	(0.007)

OR: odds ratio; CI: confidence interval; G1: 31-40 years; G2: 41-50 years; model 1: adjusted for BMI, WC, and WHR; model 2: adjusted for MDA and oxLDL; model 3: adjusted for IL-6, IL-1*β*, TNF-*α*, and MCP-1; model 4: adjusted for MDA, oxLDL, IL-6, IL-1*β*, TNF-*α*, and MCP-1; BMI: body mass index; WC: waist circumference; WHR: waist-hip ratio; MDA: malondialdehyde; oxLDL: oxidized LDL; IL-6: interleukin-6; IL-1*β*: interleukin- 1*β*; TNF-*α*: tumor necrosis factor *α*; MCP-1: monocyte chemoattractant protein-1; CDKN2A: cyclin-dependent kinase inhibitor 2A.

## Data Availability

The data used to support the findings of this study are included within the article.

## Supplementary Materials

Supplementary Materials Table S1: demographic characteristics and medications in study subjects. Table S2: oxidative stress, proinflammatory cytokines, and senescence markers in T2DM patients according to the duration of diabetes (years). Table S3: oxidative stress, proinflammatory cytokines, and senescence markers in T2DM patients according to the glycemia.
