# Supplementary material for: Middle-Aged Indians with Type 2 Diabetes Are at Higher Risk of Biological Ageing with Special Reference to Serum CDKN2A
Source: J Diabetes Res. 2020 Mar 23;2020:7569259. doi: 10.1155/2020/7569259 (PMC7128035; doi:10.1155/2020/7569259)
Supplement: Supplementary Materials — Table S1: demographic characteristics and medications in study subjects. Table S2: oxidative stress, proinflammatory cytokines, and senescence markers in T2DM patients according to the duration of diabetes (years). Table S3: oxidative stress, proinflammatory cytokines, and senescence markers in T2DM patients according to the glycemia. [file 7569259.f1.docx]

**Supplementary material**

Table S1: Demographic characteristics and medications in study subjects

| **Variables** | **G1 controls** | **G1 T2DM** | **p-value^#^** | **G2 controls** | **G2 T2DM** | **p-value** |
| --- | --- | --- | --- | --- | --- | --- |
| Gender  i) Male  ii) Female | 23 (57.5%)  17 (42.5%) | 26 (65%)  14 (35%) | 0.491 | 16 (40%)  24 (60%) | 26 (65%)  14 (35%) | 0.025 |
| Family history  i) Diabetes  ii) Diabetes and HTN  iii) HTN  iv) None | 6 (15%)  3 (7.5%)  0  31 (77.5%) | 26 (65%)  2 (5%)  1 (2.5%)  11 (27.5%) | <0.001 | 7 (17.5%)  2 (5%)  0  31 (77.5%) | 28 (70%)  4 (10%)  0  8 (20%) | <0.001 |
| OHA medications  i) Metformin  ii) Non-metformin  iii) Combinations^a^  iv) Not taken | -  -  -  - | 10 (25%)  5 (12.5%)  22 (55%)  3 (7.5%) | - | -  -  -  - | 8 (20%)  4 (10%)  27 (67.5%)  1 (2.5%) | - |
| Statin/Aspirin  i) Statin  ii) Aspirin  iii) Both^b^  iv) Not taken | -  -  -  - | 6 (15%)  3 (7.5%)  10 (25%)  21 (52.5%) | - | -  -  -  - | 4 (10%)  8 (20%)  12 (30%)  16 (40%) | - |

p-value using Chi-square test or Fischer exact test; Categorical variables represented in count (% frequency); HTN: hypertension; OHA: oral hypoglycemic agent; ^a^both metformin and other classes of OHA; ^b^statin and aspirin;

Table S2: Oxidative stress, pro-inflammatory cytokines, and senescence markers in T2DM patients according to the duration of diabetes (years)

| **Variables** | **Duration of diabetes** | | | **p-value** |
| --- | --- | --- | --- | --- |
|  | **< 2 years (n=27)** | **2-5 years**  **(n=22)** | **> 5 years**  **(n=31)** |  |
| MDA  (µmol/L) | 8.64  (6.97-10.97) | 8.1  (5.65-9.77) | 7.9  (5.72-9.96) | 0.373 |
| oxLDL  (µg/mL) | 1.1  (0.71-1.85) | 0.89  (0.69-1.7) | 1.03  (0.52-1.68) | 0.694 |
| IL-6  (pg/mL) | 3.81  (2.8-6.59) | 4.21  (2.25-4.96) | 4.88  (3.6-5.68) | 0.407 |
| IL-1β  (pg/mL) | 1.1  (0.26-2.3) | 1.08  (0.22-1.66) | 1.17  (0.83-1.94) | 0.618 |
| TNF-α  (pg/mL) | 1.36  (0-2.04) | 0.97  (0.19-1.32) | 1.1  (0.55-1.59) | 0.370 |
| MCP-1  (pg/mL) | 43.58  (23.61-112.64) | 103.15  (56.29-179.88) | 139.08  (99.63-163.99) | 0.002 |
| CDKN2A (ng/mL) | 2.9  (2.26-5.57) | 5.63  (2.86-11.76) | 7.65  (4.78-15.16) | <0.001 |

p-value using Kruskal-Wallis test; MDA: malondialdehyde; oxLDL: oxidized LDL; IL-6: interleukin-6; IL-1β: interleukin- 1β; TNF-α: tumor necrosis factor- α; MCP-1: monocyte chemoattractant protein-1; CDKN2A: cyclin-dependent kinase inhibitor 2A;

Table S3: Oxidative stress, pro-inflammatory cytokines, and senescence markers in T2DM patients according to the glycemia

| **Variables** | **HbA_1c_ (%)** | | **p-value** |
| --- | --- | --- | --- |
|  | **≤ 7.5%** | **> 7.5%** |  |
| MDA (µmol/L) | 7.9 (6.17-10.31) | 8.19 (6.84-9.83) | 0.908 |
| oxLDL (µg/mL) | 1.1 (0.64-1.67) | 1.03 (0.59-2.04) | 0.746 |
| IL-6 (pg/mL) | 3.98 (2.44-5.56) | 4.42 (2.81-5.94) | 0.596 |
| IL-1β (pg/mL) | 1.09 (0.27-1.59) | 1.19 (0.46-2.13) | 0.336 |
| TNF-α (pg/mL) | 1.0 (0.1-1.6) | 1.21 (0.35-1.58) | 0.775 |
| MCP-1 (pg/mL) | 97.57 (25.39-156.95) | 107.67 (61.66-168.12) | 0.159 |
| CDKN2A (ng/mL) | 3.99 (2.56-7.29) | 7.58 (3.64-15.02) | 0.020 |

p-value using Mann-Whitney U-test; MDA: malondialdehyde; oxLDL: oxidized LDL; IL-6: interleukin-6; IL-1β: interleukin- 1β; TNF-α: tumor necrosis factor- α; MCP-1: monocyte chemoattractant protein-1; CDKN2A: cyclin-dependent kinase inhibitor 2A;
